# Praziquantel Pretreatment Reduces *Schistosoma japonicum* Infection in Mice by Targeting Immature Worm Stages

**DOI:** 10.3390/tropicalmed10090262

**Published:** 2025-09-12

**Authors:** Xiang Gui, Rongxue Lv, Haoran Zhong, Hao Li, Ke Lu, Zhiqiang Fu, Yamei Jin, Jinming Liu

**Affiliations:** National Reference Laboratory for Animal Schistosomiasis, Key Laboratory of Animal Parasitology of Ministry of Agriculture and Rural Affairs, Shanghai Veterinary Research Institute, Chinese Academy of Agricultural Sciences, Shanghai 200241, China; grayson0222@163.com (X.G.); lvrongxue1227@163.com (R.L.); haoranzhong@shvri.ac.cn (H.Z.); lihao@shvri.ac.cn (H.L.); luke@shvri.ac.cn (K.L.); fuzhiqiang@shvri.ac.cn (Z.F.); yameijin@shvri.ac.cn (Y.J.)

**Keywords:** schistosomiasis, *Schistosoma japonicum*, praziquantel, pretreatment, delayed infections

## Abstract

Schistosomiasis remains a significant public health concern, with *Schistosoma japonicum* infection endemic in certain regions of Asia. Praziquantel (PZQ), primarily known as an effective therapeutic agent, has recently shown potential as a prophylactic measure against delayed *S. japonicum* infections. This study investigated the preventive efficacy of PZQ pretreatment at varying cercarial infection intensities and determined the parasite developmental stages targeted by the pretreatment in a mouse model. Results demonstrated that PZQ pretreatment significantly reduced both worm burdens and liver egg counts at low (10 and 20 cercariae) and high (100 cercariae) infection intensities, with reductions in worm burdens ranging from approximately 48% to 60% and liver egg counts by 47% to 73% compared to control groups (*p* < 0.05). Further analysis revealed that the mortality of parasites in PZQ-pretreated mice predominantly occurred during the juvenile schistosomula stages, particularly in worms younger than 15 days post-infection. These findings provide critical evidence supporting the application of PZQ pretreatment as a practical prophylactic measure to prevent *S. japonicum* infections, particularly in populations and animals frequently exposed to contaminated water in endemic areas.

## 1. Introduction

Schistosomiasis is a globally prevalent parasitic disease that imposes a significant burden on both public health and economic development [1]. Among the *Schistosoma* species that most severely affect humans, *Schistosoma japonicum* remains endemic in several regions, particularly in China, the Philippines, and Indonesia [2]. Infection with *S. japonicum* can lead to serious pathological consequences in the host, including granulomatous inflammation, chronic immune activation, and progressive hepatic fibrosis [3]. These outcomes pose major threats to both human and animal health and hinder sustainable socio-economic development in endemic areas [4].

Since the introduction of praziquantel (PZQ) in the late 1970s, significant progress has been made in the global control of schistosomiasis [5]. However, in several endemic regions, the prevalence of *S. japonicum* infection remains alarmingly high in both humans and domestic animals [6]. In 2022, the World Health Organization (WHO) released updated guidelines focused on the control and elimination of human schistosomiasis, recognizing it as a major public health threat [7]. Given this context, there is an urgent need to develop new vaccines or chemoprophylactic agents to effectively combat schistosomiasis in both humans and domestic ruminants, which serve as key reservoirs for zoonotic transmission.

PZQ is a broad-spectrum anthelmintic that exhibits high efficacy against various *Schistosoma* species in both human and veterinary applications [8]. While the precise mechanism of action remains incompletely understood, studies suggest that PZQ disrupts calcium ion homeostasis in the parasite by modulating voltage-gated calcium channels, resulting in elevated intracellular Ca^2+^ levels, tegumental damage, and enhanced recognition and clearance by the host immune system [9,10,11]. However, due to its rapid metabolism in mammalian hosts, PZQ has traditionally been considered a therapeutic agent rather than a prophylactic one, with limited protective efficacy against reinfection [12].

Interestingly, recent findings indicate that PZQ, when administered at appropriate doses, can effectively prevent delayed infection with *S. japonicum* [13]. It has been demonstrated that pretreatment of mice with oral PZQ at a dose of 300 mg/kg (administered twice) or a single equivalent injection provides protection against moderate-dose *S. japonicum* infection, with a protective window lasting up to 18 days [13]. Furthermore, the prophylactic effect of PZQ in key reservoir hosts, including buffaloes and goats, has been confirmed, with significant reductions in worm burden observed at doses of 13 mg/kg and 25 mg/kg, respectively [14]. These results highlight the potential of PZQ not only as a therapeutic agent but also as a preventive intervention capable of reducing parasite transmission between livestock and humans.

To further validate the preventive efficacy of PZQ against delayed *S. japonicum* infection and to provide practical strategies for schistosomiasis control in low-, moderate-, and high-endemic regions, the present study investigates the effects of PZQ pretreatment at varying doses on cercarial challenge in a mouse model. Additionally, the key developmental stages during which parasite mortality occurs following pretreatment are examined. This study aims to provide novel scientific evidence supporting the preventive application of PZQ and to inform future public health interventions aimed at controlling schistosomiasis transmission.

## 2. Materials and Methods

### 2.1. Ethical Considerations

Ethical approval for the animal experiments reported in this manuscript was obtained from the Shanghai Veterinary Research Institute’s “Animal Care and Use Committee”: Permit number: SHVRI-SZ-20200427-01.

### 2.2. Animals, Parasites, and Drugs

The *S. japonicum* Anhui strain used in this study was maintained in our laboratory. Freshly shed cercariae were prepared by exposing infected snails to light. Light microscopy was used to determine the numbers and the viability of cercarial prior to infection. Specific pathogen free male BALB/c mice, six-to-eight-week of age, were purchased from Jiesijie Laboratory Animal Co., Ltd., Shanghai, China. The PZQ was purchased from Hubei Aibo Technology Co., Ltd. (Xianning, China) and prepared as an oral formulation by dissolving the drug in a 1% carboxymethyl cellulose solution before administration.

### 2.3. PZQ Pretreatment, and Challenge Infection

Two independent trials were conducted to evaluate the protective efficacy of PZQ pretreatment. Trial 1 aimed to assess the prophylactic effect of PZQ pretreatment against cercarial infection at different challenge doses (10, 20, and 100 cercariae). BALB/c mice were randomly divided into six groups (*n* = 8 per group), including PZQ-pretreated groups (10, 20, and 100 cercariae) and corresponding control groups (10, 20, and 100 cercariae). Mice in the PZQ-pretreated groups received oral administration of PZQ (300 mg/kg) on days-16 and -15, with a 24 h interval [13]. Control group mice were administered 1% carboxymethyl cellulose solution at the same time points. On day 0, mice in each group were challenged with 10, 20, or 100 cercariae, according to their assigned group.

Trial 2 was designed to identify the target stage of the protective effect. The trial was independently repeated twice. In each repetition, BALB/c mice were randomly assigned to two groups: a PZQ-pretreated group and a control group, with 25 mice per subgroup. PZQ-pretreated mice received the same oral treatment schedule (24 h apart, on days-16 and -15) and dosage (300 mg/kg) that PZQ-pretreated mice in trial 1 received [13]. Control mice received 1% carboxymethyl cellulose solution at the same time points. On day 0, all mice were challenged with 100 cercariae. At 5, 10, 15, 22, and 42 days post-infection (dpi), 5 mice from each group were randomly selected and sacrificed for further analysis.

### 2.4. Worm Burdens and Liver Egg Counting Assessment

All mice in Trial 1 were sacrificed on 42 dpi. Adult worms were recovered and counted via hepatic portal vein perfusion. The liver was also collected to assess egg burden. Briefly, mice were euthanized at the designated time points, and the hepatic portal vein was exposed. A 21-gauge needle was inserted into the portal vein, and warm saline (37 °C) was perfused at a constant flow rate until the liver and mesenteric veins were cleared of blood. The effluent was collected in a glass dish, and the adult schistosomes were carefully retrieved under a stereomicroscope and counted. For liver egg quantification, a portion of the liver (approximately 0.5–1 g) was weighed and homogenized in 5 mL of 10% potassium hydroxide (KOH). The homogenates were incubated overnight at 37 °C to dissolve the tissue. The number of eggs was counted under a light microscope, and the results were expressed as eggs per gram (EPG) of liver tissue.

In Trial 2, mice were sacrificed at designated time points (days 5, 10, 15, 22, and 42 dpi) to collect worms at corresponding developmental stages. With the exception of 5-day-old schistosomula, worms at all other time points were recovered from the hepatic portal vein by perfusion and subsequently counted. Five-day-old schistosomula were collected using the following procedure: mouse lungs were minced into small pieces and incubated in Dulbecco’s Modified Eagle Medium (Gibco, Shanghai, China) supplemented with 10% fetal calf serum (Dogesce, Beijing, China) at 37 °C with 5% CO_2_. After the schistosomula emerged from the tissue, the lung fragments were removed and red blood cell lysis buffer (Tiangen, Shanghai, China) was added according to the manufacturer’s instructions. After 5 min of incubation, schistosomula were counted under a microscope.

### 2.5. Data Analysis

Data were analyzed using Statistical Package for the Social Sciences (Version 27.0, SPSS software, Chicago, IL, USA) and GraphPad Prism (Version 9.0, Grappa Software, San Diego, CA, USA) and were presented as the mean ± standard deviation (SD). Statistical differences were determined using one-way analysis of variance and *p* values < 0.05 were considered to be statistically significant.

## 3. Results

### 3.1. PZQ Pretreatment Significantly Reduces Worm Burdens and Liver Egg Counts at Various Infection Intensities

**Figure 1:**
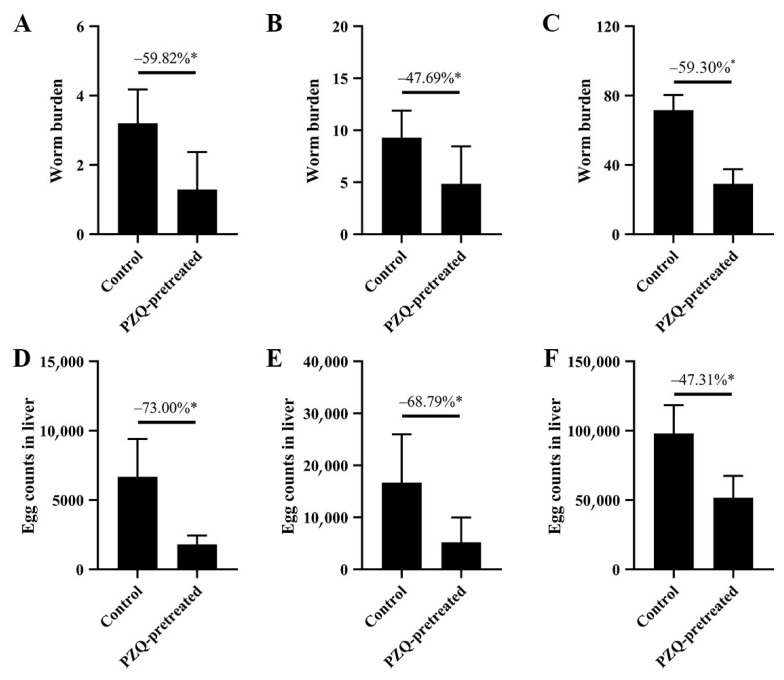
PZQ pretreatment significantly reduces worm burdens and liver egg counts in mice under various infection intensities. BALB/c mice were pretreated with oral PZQ at a dose of 300 mg/kg on days-16 and -15 before being challenged with cercariae at different intensities ((**A**,**D**): 10 cercariae; (**B**,**E**): 20 cercariae; (**C**,**F**): 100 cercariae). At 42 dpi, worm burdens (**A**–**C**) and liver egg counts (expressed as eggs per gram of liver, EPG; (**D**–**F**)) were measured. Bars represent mean ± SD. Percent reductions compared with the respective control groups are indicated. * *p* < 0.05, statistically significant compared with control.

The results from Trial 1 demonstrated that, at challenge doses of 10, 20, and 100 cercariae, the worm burdens in the PZQ-pretreated group at 42 dpi were 1.29 ± 1.48, 4.86 ± 3.60, and 29.17 ± 8.39, respectively. These values were reduced by 59.82%, 47.69%, and 59.30%, compared to the respective control groups (Figure 1A–C). The liver egg counts in the PZQ-pretreated groups were 1804.46 ± 2640.11, 5205.82 ± 4783.51, and 51,695.69 ± 15,719.64, with reductions of 73.00%, 68.79%, and 47.31%, respectively, in comparison to the control groups (Figure 1D–F).

### 3.2. Target Stages of Schistosome Mortality in PZQ-Pretreated Mice

**Figure 2:**
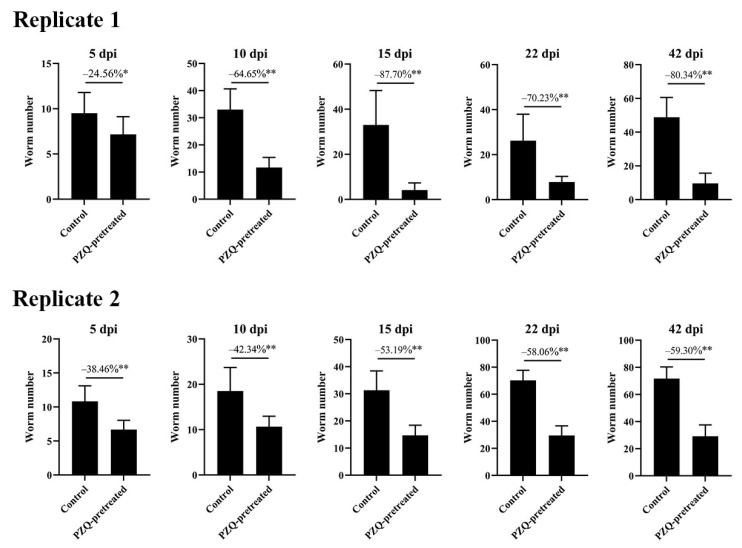
Schistosome mortality primarily occurs at juvenile stages in PZQ-pretreated mice. Two independent experimental replicates were conducted, each involving BALB/c mice pretreated with oral PZQ at a dose of 300 mg/kg on days-16 and -15, followed by challenge with 100 cercariae. Worm numbers were quantified at indicated time points (5, 10, 15, 22, and 42 dpi). Bars represent mean ± SD. Percent reductions relative to the respective control groups are indicated. * *p* < 0.05, ** *p* < 0.01, statistically significant compared with control.

Two independent experimental replicates were conducted in Trial 2 to determine the target stages of schistosome mortality in PZQ-pretreated mice. Consistent results were obtained across both replicates. Compared to the respective control groups, the number of schistosome worms collected at various developmental stages was significantly reduced in the PZQ-pretreated groups in both replicates. The reduction in the number of worms at 5, 10, and 15 dpi progressively increased in both replicates, from 24.56% (Replicate 1) and 38.46% (Replicate 2) to 87.70% (Replicate 1) and 53.19% (Replicate 2). The reduction rates for worms at 15 dpi, 22 dpi, and 42 dpi remained relatively stable (Figure 2). The data indicate that while some reductions in worm numbers were observed at 5 and 10 dpi, the most pronounced decreases occurred from 15 dpi onward, suggesting that the principal effect of PZQ pretreatment is exerted on juvenile parasites at or beyond this stage.

## 4. Discussion

The intensity of *S. japonicum* transmission varies across different endemic regions due to variations in topography, climate, and control measures [2]. In China, thanks to the strong commitment of the government and its effective implementation of comprehensive control strategies, the prevalence and intensity of *S. japonicum* infection in both livestock and humans have significantly decreased [15]. By 2024, the infection rates and worm burdens of *S. japonicum* have been reduced to low levels, and the National Schistosomiasis Control Program has entered the elimination phase [16]. However, despite successful control efforts in many regions, feasible preventive measures are still lacking. Some areas with poorer environmental conditions continue to report positive cases with high prevalence rates. For instance, in the Philippines, a study by Mario Jiz et al. [6] found that water buffaloes in a naturally exposed region had an infection rate as high as 97%, with worm burdens ranging from 49 to 138, averaging 94 worms. Thus, aside from traditional control measures focused on reducing the intermediate snail host and avoiding contact with contaminated water, it is also critical to develop preventive approaches, such as drug administration or vaccination, for individuals and animals at risk of exposure [17,18].

In this context, a relatively low infection dose (10–20 cercariae) was employed in the present study to better simulate the exposure levels typically observed in low-endemic areas. It is acknowledged that a proportion of cercariae (approximately 30–40%) may be cleared by the host immune system, which could have influenced the recovery data. This limitation has been noted, and the results should therefore be interpreted with this consideration in mind.

Previous studies have demonstrated that PZQ-pretreatment can prevent subsequent moderate-dose cercarial infections in a mouse model, establishing the effective dose, duration of protection, and onset of efficacy [13]. In this study, we confirmed that PZQ pretreatment significantly reduced worm burdens and liver egg counts in mice infected with low (10 or 20 cercariae) and high (100 cercariae) doses of *S. japonicum* (*p* < 0.05). Additionally, we have shown that PZQ is effective in preventing subsequent infections in key reservoir hosts, such as water buffaloes and goats [14]. These findings suggest that PZQ-pretreatment could serve as a practical preventive measure against *S. japonicum* infection in regions with low, moderate, or high endemicity, especially for individuals and animals that are exposed to contaminated water. However, it is not intended to imply that mass drug administration (MDA) should be implemented at impractically short intervals (e.g., every 18 days). Rather, our results provide experimental evidence of a prophylactic window, which may inform integrated strategies and highlight possible benefits in specific high-risk populations.

Upon entering the human or animal host, *S. japonicum* cercariae transform into schistosomula, then migrate to the liver and pair by day 15 post-infection. The adult worms migrate to the mesenteric veins around day 24, where they produce eggs [3]. To further explore the mechanism of PZQ in preventing schistosome infection, we observed the differences in worm counts between PZQ-pretreated and control mice at 5, 10, 15, 22, and 42 dpi. Decreases in the number of 5, 10, and 15-day-old worms progressively increased with time from 24.56 to 38.46% through to 53.19–87.70%. The reduction rates for worms at 15, 22, and 42 days remained stable. These data suggest that the most pronounced effects of PZQ pretreatment were observed from 15 dpi onward, indicating that the drug is more effective against relatively mature juvenile stages rather than the earliest schistosomula. This is in contrast to PZQ’s mechanism of action against adult worms, which is well-documented. PZQ is known to effectively kill adult schistosomes, but it is less effective against schistosomula [19,20]. Previous research suggests that PZQ eliminates 90% of adult worms within 24 h of oral administration [13]. Therefore, the preventive effects observed in the PZQ-pretreated mice are not due to direct inhibition of the schistosomes but rather a preemptive mechanism that likely involves the early stages of the parasite’s lifecycle. Further research is required to fully understand the exact mechanisms by which PZQ pretreatment prevents schistosomiasis in these models.

## Data Availability

The original contributions presented in this study are included in the article. Further inquiries can be directed to the corresponding author.
